# Long-Term Follow-up of Gastric Precancerous Lesions in a Low GC Incidence Area

**DOI:** 10.14309/ctg.0000000000000237

**Published:** 2020-12-03

**Authors:** Nicolas Chapelle, Matthieu Péron, Lucille Quénéhervé, Alice Bourget, Maxime Leroy, Yann Touchefeu, Estelle Cauchin, Emmanuel Coron, Jean François Mosnier, Tamara Matysiak-Budnik

**Affiliations:** 1 CHU de Nantes, Hôtel Dieu, Institut des Maladies de l'Appareil Digestif, Nantes, France;; 2 Université de Nantes, Nantes, France;; 3 Centre de Recherche en Transplantation et Immunologie (CRTI), UMR 1064, INSERM, Nantes, France;; 4 INSERM U 1235, The Enteric Nervous System in Gut and Brain Disorders, Nantes, France;; 5 Département de Biostatistiques, CHU de Nantes, Nantes, France;; 6 Service d'Anatomie Pathologique, CHU de Nantes, Nantes, France.

## Abstract

Data on the evolution of gastric precancerous lesions (GPL), especially in countries of a Low gastric cancer incidence area are limited. Our objective was to study a long-term evolution of GPL in France.

**METHODS:**

All the patients diagnosed with GPL (atrophic gastritis, intestinal metaplasia [IM], and dysplasia) between 2000 and 2015 and fulfilling criteria for evolution assessment (at least 2 endoscopies, minimal follow-up of 6 months, and at least 2 biopsies obtained from the antrum and corpus) were included. Clinical and endoscopic data were analyzed, and histological samples were reviewed by an expert pathologist with evaluation of the Operative Link on Gastric Intestinal Metaplasia Assessment stage and type of IM.

**RESULTS:**

From the 507 patients with GPL, 79 fulfilled the strict criteria. During a mean follow-up of 66 months, during which the patients had a mean number of 4 endoscopies (min–max: 2–21) with 9 biopsies/endoscopy, a stability was observed in 70% of patients. Progression occurred in 14% of patients, within a mean delay of 62.1 months (min–max: 17–99). Progression of the lesions was significantly higher in patients with incomplete type of IM (relative risk of progression for incomplete IM: 11.5; 95% confidence interval 2.5–53.1). Regression of IM occurred in 16% of the patients, after a mean delay of 90 months.

**DISCUSSION:**

This study shows that the patients with antrum-limited IM, especially of incomplete type, are at the highest risk of developing gastric cancer. In most patients, however, the lesions remain stable, which highlights the need for additional markers to better target the patients at risk of progression.

## INTRODUCTION

Despite constantly decreasing incidence (1,2), gastric cancer (GC) is the fifth most common cancer in the world and the third leading cause of cancer-related death (3). Its prognosis remains poor, with a 5-year overall survival inferior to 30%–40% (4,5). This prognosis is closely related to the stage of the disease at diagnosis, being good in early stages (6), and very poor at advanced stages of the disease. Early detection of GC appears as the best strategy to reduce GC-related mortality. Indeed, the best survival rates in GC are observed in the East Asian countries, such as Korea or Japan, where efficient endoscopic cancer screening programs have been developed (5,7–9).

Gastric carcinogenesis is a multistep process, known as Correa's cascade (10,11), beginning with chronic gastritis usually induced by infection with *Helicobacter pylori*, which may evolve into gastric precancerous lesions (GPL), i.e., successively, into atrophic gastritis (AG), intestinal metaplasia (IM), low-grade dysplasia (LGD), high-grade dysplasia (HGD), and eventually adenocarcinoma. During this process, the role of *H. pylori* infection, recognized since 1994 as a class A carcinogen by the World Health Organization (12), acting as its initiator through the induction of gastric inflammation, and on its evolution toward GC, is of major importance. The surveillance of patients with GPL appears as a logical approach to reduce the mortality from GC, and several European guidelines on management and surveillance of these patients have been recently published (13–16). In overall, these guidelines recommend the surveillance of advanced GPL (i.e., AG and/or IM extended into the antrum and corpus), while the indications for surveillance of antrum-limited GPL are considered less clear and depend on individual patient's characteristics and specific characteristics of the lesions. The data on the evolution of GPL in the countries of low GC incidence such as France (5) are limited. Therefore, our aim was to analyze the evolution of GPL in a long-term follow-up study in France.

## PATIENTS AND METHODS

All the patients who underwent an upper digestive endoscopy with gastric biopsies between January 1, 2000, and December 31, 2015, in the University Hospital of Nantes, France, and in whom a GPL was diagnosed, were identified within the hospital pathology database. The patients with the following GPL (by order of increasing severity) were included: AG, IM, LGD, and HGD. All the data, including demographic and clinical data, histological data (type and localization of GPL, presence of *H. pylori*), and the follow-up data (evolution of GPL), were collected from the medical files and analyzed retrospectively.

From this cohort of patients, to provide results using only high-quality data and to analyze accurately the evolution of GPL over the time, we selected the patients fulfilling the following strict criteria: (i) at least 2 endoscopies performed at a minimal interval period of 6 months, (ii) at each endoscopy, at least 2 biopsies obtained from the antrum and 2 from the corpus, and (iii) histology material available for a prospective review of the biopsy specimens. Patients who did not fulfill these criteria, as well as those in whom GPL were found on macroscopic visible lesions (mostly adenomas) removed by endoscopic resection, were excluded from the analysis to focus on the natural evolution of GPL of “flat mucosa.”

The biopsy specimens of these patients were retrieved from the hospital tissue bank and reanalyzed by an expert pathologist. All the biopsy specimens were analyzed for the presence of GPL and their extent according to Operative Link Gastritis Assessment and Operative Link on Gastric Intestinal Metaplasia (OLGIM) scores and the presence of *H. pylori* using Giemsa stain. All specimens were scored according to the updated Sydney classification (17). For each biopsy sample, *H. pylori* density, acute inflammation (neutrophil infiltration), chronic inflammation (mononuclear cell infiltration), AG, and IM were assessed (0 = absent, 1 = mild, 2 = moderate, and 3 = marked). Dysplasia was scored according to the Vienna system, which includes LGD and HGD (18,19). The OLGIM system was used to evaluate the severity and distribution of IM in the stomach (20). IM was categorized as of complete or incomplete type, and the biopsy specimens showing both types were categorized as incomplete.

All the patients with confirmed *H. pylori* infection received eradication treatment, using different regimes varying according to the period. Eradication status was evaluated by histology and/or urea breath test.

### Statistical analysis

Statistical analysis was performed using the SAS (statistical analysis system) software (SAS Institute Inc., Cary, NC). Chi-squared or Fisher tests were used for qualitative data, Student or Wilcoxon-Mann-Whitney tests were used for quantitative data analysis, and the McNemar test was used for paired data. Univariate logistic regression was applied to determine relative risks of progression. The distribution of values was presented either by the mean (+/−SD), or, in case of wide variation, by the median (with the first and third quartile Q1; Q3).

The study was approved by the local ethical committee.

## RESULTS

### Patients characteristics at inclusion

Demographic, endosocopic, and histologic characteristics of the cohort at baseline have been decribed elsewhere (21). Briefly, among the 16,764 patients who underwent an upper endoscopy with biopsies for any reason, 507 patients with a GPL were identified, and 254 of them have had at least 2 upper endoscopies during the study period. Details of the histological findings between first and last endoscopy are provided in supplementary materials (see Supplementary Table 1, Supplementary Digital Content 3, http://links.lww.com/CTG/A435). Among them, 140 patients were considered to have a “follow-up endoscopy” (i.e., performed within a minimal delay of 6 months between 2 examinations). Finally, after applying the other strict criteria, the histological samples of 79 patients (35 men, median age: 61 years [min–max: 29–84 years]) were retained for analysis. Clinical characteristics of the patients are presented in Table 1. The mean (±SD) follow-up duration was 66 (±48) months (min = 7, max = 208), and each patient had a mean (±SD) of 4 (±2) endoscopies (min = 2, max = 14) during the follow-up period with a mean (±SD) number of 9 (±4) biopsies (min = 4, max = 21) per endoscopy. In total, the results of 341 endoscopies were reanalyzed. Histologic characteristics of the patients at initial and final endoscopy are detailed in Table 2. At first endoscopy, most of the patients had IM (92%) of complete type (82%) and located to the antrum only (81%). Regarding OLGIM, patients were classified OLGIM I (53%), OLGIM II (39%), and OLGIM III (8%), but none of them was staged OLGIM IV at baseline.

**Table 1.**
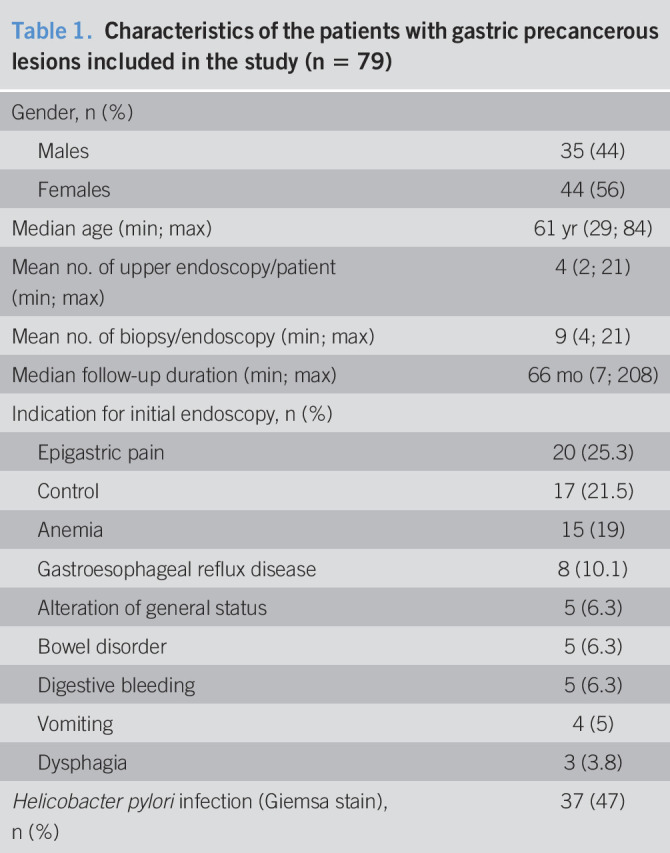
Characteristics of the patients with gastric precancerous lesions included in the study (n = 79)

Gender, n (%)	
Males	35 (44)
Females	44 (56)
Median age (min; max)	61 yr (29; 84)
Mean no. of upper endoscopy/patient (min; max)	4 (2; 21)
Mean no. of biopsy/endoscopy (min; max)	9 (4; 21)
Median follow-up duration (min; max)	66 mo (7; 208)
Indication for initial endoscopy, n (%)	
Epigastric pain	20 (25.3)
Control	17 (21.5)
Anemia	15 (19)
Gastroesophageal reflux disease	8 (10.1)
Alteration of general status	5 (6.3)
Bowel disorder	5 (6.3)
Digestive bleeding	5 (6.3)
Vomiting	4 (5)
Dysphagia	3 (3.8)
*Helicobacter pylori* infection (Giemsa stain), n (%)	37 (47)

**Table 2.**
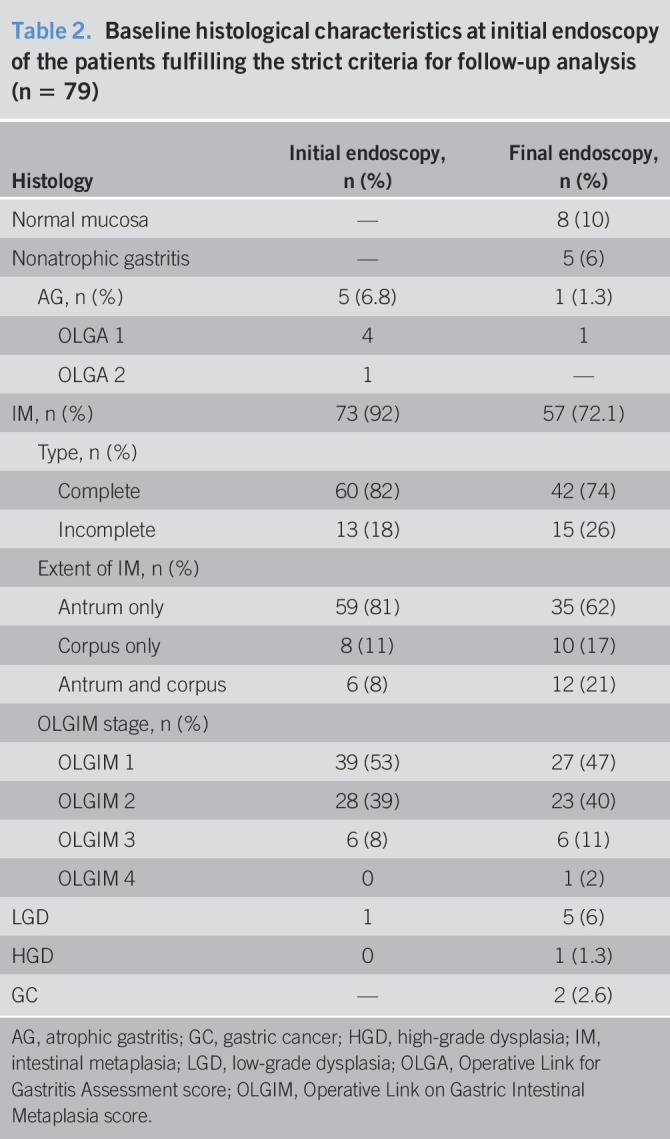
Baseline histological characteristics at initial endoscopy of the patients fulfilling the strict criteria for follow-up analysis (n = 79)

Histology	Initial endoscopy, n (%)	Final endoscopy, n (%)
Normal mucosa	—	8 (10)
Nonatrophic gastritis	—	5 (6)
AG, n (%)	5 (6.8)	1 (1.3)
OLGA 1	4	1
OLGA 2	1	—
IM, n (%)	73 (92)	57 (72.1)
Type, n (%)		
Complete	60 (82)	42 (74)
Incomplete	13 (18)	15 (26)
Extent of IM, n (%)		
Antrum only	59 (81)	35 (62)
Corpus only	8 (11)	10 (17)
Antrum and corpus	6 (8)	12 (21)
OLGIM stage, n (%)		
OLGIM 1	39 (53)	27 (47)
OLGIM 2	28 (39)	23 (40)
OLGIM 3	6 (8)	6 (11)
OLGIM 4	0	1 (2)
LGD	1	5 (6)
HGD	0	1 (1.3)
GC	—	2 (2.6)

AG, atrophic gastritis; GC, gastric cancer; HGD, high-grade dysplasia; IM, intestinal metaplasia; LGD, low-grade dysplasia; OLGA, Operative Link for Gastritis Assessment score; OLGIM, Operative Link on Gastric Intestinal Metaplasia score.

Overall, a stability of GPL was observed in 55 patients (70%), a progression to more severe lesion in 11 patients (14%, including 2 patients with gastric adenocarcinoma), and a regression in 13 patients (16%, including 8 to normal gastric mucosa and 5 to non-AG). Altogether, the progression was observed after a mean delay of 62.1 months (min–max: 17–99). The 2 patients (2.6%) who progressed to GC presented initially antrum-limited incomplete IM (OLGIM II and III) and had a delay 85 and 99 months, respectively, between first endoscopy and diagnosis of cancer. The patient in whom progression to HGD was observed after a delay of 17 months, presented initially an antrum-limited incomplete IM OLGIM III. In all the patients who regressed into normal mucosa, none had OLGIM III or IV stages. Evolution according to OLGIM stage was not significant (*P* = 0.5271). Precise evolution of the patients is shown in Table 3.

**Table 3.**
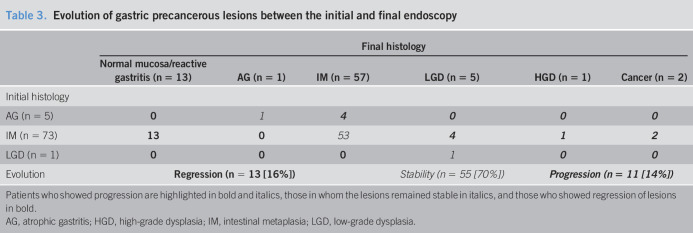
Evolution of gastric precancerous lesions between the initial and final endoscopy

	Final histology					
	Normal mucosa/reactive gastritis (n = 13)	AG (n = 1)	IM (n = 57)	LGD (n = 5)	HGD (n = 1)	Cancer (n = 2)
Initial histology						
AG (n = 5)	**0**	*1*	***4***	***0***	***0***	***0***
IM (n = 73)	**13**	**0**	*53*	***4***	***1***	***2***
LGD (n = 1)	**0**	**0**	**0**	*1*	***0***	***0***
Evolution	**Regression (n = 13 [16%])**			*Stability (n = 55 [70%])*	***Progression (n = 11 [14%])***	

Patients who showed progression are highlighted in bold and italics, those in whom the lesions remained stable in italics, and those who showed regression of lesions in bold.

AG, atrophic gastritis; HGD, high-grade dysplasia; IM, intestinal metaplasia; LGD, low-grade dysplasia.

As far as the evolution of IM according to the OLGIM score is concerned, the stability, regression, and progression were, respectively, observed in 41%, 23%, and 36% of patients with OLGIM I, in 28%, 36%, and 36% in patients with OLGIM II, and in 0%, 67%, and 33% in OLGIM III.

According to the type of IM, of 13 patients with incomplete IM, 5 (38%) showed progression, as compared to only 2 of 60 patients (3%) with complete type of IM (*P* = 0.0015). The relative risk of progression in patients with incomplete IM was 11.5 (95% confidence interval 2.5–53.1). All the 7 patients in whom a progression from IM to more severe lesion was observed had initially antrum-limited IM, including 5 of them (71%), who had IM of incomplete type. Conversely, all the patients who showed a regression had a complete type of IM. Among the 60 patients with complete type of IM, 13 (22%) showed a regression of IM during the follow-up, while none of the 13 patients with incomplete type of IM showed regression (*P* = 0.06). The disappearance of IM was observed in these patients after a median follow-up period of 90 months (min = 7, max = 147). From 73 patients with IM at initial endoscopy, the OLGIM stage remained stable in 29 patients (40%), the OLGIM stage decreased in 23 patients (31%), and in 21 patients (29%), the OLGIM stage increased. The detailed analysis of IM evolution according to OLGIM stage is presented in Table 4.

**Table 4.**
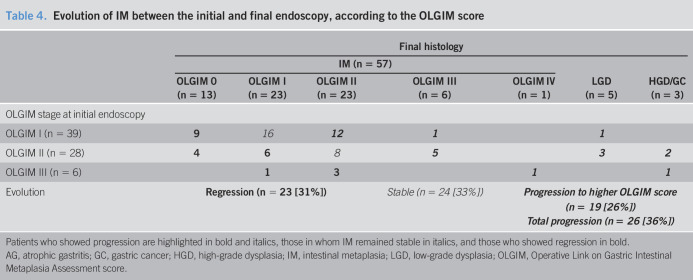
Evolution of IM between the initial and final endoscopy, according to the OLGIM score

	Final histology						
	IM (n = 57)					LGD (n = 5)	HGD/GC (n = 3)
	OLGIM 0 (n = 13)	OLGIM I (n = 23)	OLGIM II (n = 23)	OLGIM III (n = 6)	OLGIM IV (n = 1)		
OLGIM stage at initial endoscopy							
OLGIM I (n = 39)	**9**	*16*	***12***	***1***		***1***	
OLGIM II (n = 28)	**4**	**6**	*8*	***5***		***3***	***2***
OLGIM III (n = 6)		**1**	**3**		***1***		***1***
Evolution	**Regression (n = 23 [31%])**			*Stable (n = 24 [33%])*	***Progression to higher OLGIM score (n = 19 [26%])***		
					***Total progression (n = 26 [36%])***		

Patients who showed progression are highlighted in bold and italics, those in whom IM remained stable in italics, and those who showed regression in bold.

AG, atrophic gastritis; GC, gastric cancer; HGD, high-grade dysplasia; IM, intestinal metaplasia; LGD, low-grade dysplasia; OLGIM, Operative Link on Gastric Intestinal Metaplasia Assessment score.

Thirty-seven patients (47%) were *H. pylori*-positive by histology at initial endoscopy. Eradication regimen used were the following: amoxicillin + azithromycin in 12 patients, sequential triple therapy in 10 patients, bismuth quadruple therapy in 4 patients, and other regimens (rifabutin + amoxicillin, ciprofloxacin + rifampicin + minocyclin, amoxicillin + levofloxacin, and amoxicillin + clarithromycin) in 5 patients. In 5 patients, the eradication regimen was not precised, and 1 other patient died before eradication could be performed. Effective bacterial eradication was obtained after 1, 2, 3, and 4 lines of treatment in 25, 2, 8, and 1 patient, respectively. In 6 patients (7%), *H. pylori* was still present at final endoscopy. The eradication status was evaluated by histology in 16 patients and by histology + breath test in 20 patients.

## DISCUSSION

A better understanding of the GPL evolution is of key importance to establish the optimal screening and follow-up modalities for the individuals with an increased risk of developing GC. There are wide differences in terms of epidemiology and management of GPL between the high- and low GC incidence area. Most of the existing evidence comes from the first group of countries (22–28), essentially the East Asian countries, and they cannot be fully transposed to low-incidence areas like most of the European countries. However, few studies are now also available from the low-risk areas (29–33).

In our population, in 13% of the patients, GPL progressed to more severe lesions during the follow-up, including the 7 patients who developed GC. This overall progression rate is higher than that reported by den Hoed et al. (6%) (34) and by den Hollander et al. (4%) (30), but similar to that reported by Dinis-Ribeiro et al. (from 8% to 32%, depending on the type of initial lesion (24). Conversely, a regression of the GPL was found in 30% of our patients, similarly to that reported by den Hoed et al. (29) and by den Hollander et al. (30). Data obtained on such retrospective study may be biased because of several factors, including sampling error, missing data, or short time interval between 2 successive endoscopies. Therefore, to surround this bias, we secondarily applied very strict selection criteria and reanalyzed the biopsy samples to provide the reliable data on the exact evolution of the GPL. Several results obtained from this sample of patients with the strict criteria deserve to be discussed. The severity of IM as expressed by OLGIM stage appears lower than reported before, with only 8% of the patients with OLGIM III and no patient with OLGIM IV IM, as compared to 20% and 9%, respectively, in the study of den Hollander et al. (30). The proportion of incomplete IM is lower as compared to high-risk areas (35) but similar as the one reported in another low GC incidence area study (32).

As far as the evolution of GPL is concerned, we confirm that the risk of progression to GC is the most important in the patients with the highest OLGIM stages (28,36). Interestingly, both patients who progressed to GC had initially antrum-limited IM and thus were not candidates for systematic endoscopic surveillance according to the European guidelines applicable at this time (14). The question of surveillance of the patients with IM remains still debatable. Although this surveillance is formally recommended for the patients with extensive IM (13–15,37), the benefit and cost-effectiveness of such a strategy for antrum-limited IM was not clear and is not currently systematically recommended. Indeed, several studies have clearly shown that the more the IM is spread through the stomach, the greater is the risk of progression to GC (38–40). As illustrated by the 2 cases, our study shows that antrum-limited IM patients also bear a risk of progression to more severe lesions, including GC, especially in case of incomplete type of IM. This is an important information, indicating that also in the low GC incidence area such as France, the type of IM is an important parameter to be taken into account because it has been already shown in high- or intermediate-GC risk European countries such as Portugal (24–28) or in Asia (32).

The relative risk of progression in patients harboring incomplete IM found in our study is concordant with those reported by others in different, Asian, South American, or European, populations (increased risk by 6–14) (26,28,32). Our study suggests that this parameter is applicable for both low- and high-risk population. It has been argued that the diagnosis of the complete or incomplete character of the IM is difficult and not reproducible, and there seems to be a poor interobserver agreement in typing of IM. For this reason, this parameter was not included in the first management of precancerous conditions and lesions in the stomach guidelines (14) and is not recommended in practice by the recent guidelines of the British Society of Gastroenterology (13). However, an Asian study found an interobserver agreement of 85% for classifying the IM into complete or incomplete type (32). Given the importance of accumulated evidence, in the new version of management of precancerous conditions and lesions in the stomach and American Gastroenterological Association guidelines, it is stated that patients with a 1-site limited incomplete IM may benefit from the surveillance endoscopy (15,41), and our results clearly support this statement.

Another point highlighted by this study is that IM may be reversible and particularly in patients with the low OLGIM stages. Although still debated in the literature (42,43), our results show that IM should not be considered univocally as a “point of no return” in the carcinogenic process (44). Indeed, as discussed previously, beyond the term “IM,” several other factors, not taken into account in all the studies and meta-analyses, may indicate reversibility of IM (45,46), such as the OLGIM stage, the type of IM, and other molecular factors, not routinely investigated yet (47), but which should be considered. Moreover, a relatively short follow-up period of the patients included in these meta-analyses (less than 3 years in most studies included in these meta-analyses) (45,46), or in the other studies (29,32,33), was probably not long enough to detect a “true” regression. The long duration of follow-up in our study (mean of 7.5 years and maximal of 12.3 years) allowed us to see a progressive healing of the gastric mucosa.

Finally, we confirm that, even in a long-term surveillance, in the majority of patients (70%–90%), GPL remain stable over time (29,30,33). This point highlights the need to develop more accurate and personalized algorithm of surveillance of these patients to avoid an excessive surveillance and unnecessary burden to the patients and cost to the society. Combinations of several parameters (extent, type of IM, modalities of endoscopy, and patient's history and lifestyle (30,48–50)), should be considered to better identify the patients with GPL the most susceptible to benefit from adequate surveillance. The application and improvement of the guidelines (15,51) is crucial, and research should continue to target more precisely the patients “the most at-risk” among those with GPL. The widespreading use of noninvasive markers (48), the development of new screening methods (52), and the combination of interventional and screening strategies (53) will probably improve the management of patients at risk of GC. New tools such as machine learning, capable to analyzed complex algorithms with numerous parameters, are being developed and may become helpful in clinical practice in this setting in the future (54).

Our study has some limitations. First, the number of patients analyzed is relatively small as compared to some population-based or large cohort studies (30,31,33), which may probably explain the absence of significance in the differences of evolution according to OLGIM stage. The sample size and the diagnostic method (histology only) did not allow to provide information on the precise role of *H. pylori*. However, as compared to some other studies of a similar size performed in low GC incidence area our series has the longest duration of follow-up, the highest number of endoscopies per patient, and the highest number of biopsies obtained during each endoscopy, all these factors reducing significantly the risk of false diagnosis due to sampling error (29,30,32,33). Moreover, the global trends for evolution were similar in the global and in the restrictive population.

The second limitation of our study is related to the fact that most of the biopsies were taken randomly, during white-light endoscopies, and using the old devices especially during the first part of the study. We have previously shown that the predictive value of macroscopic assessment of the gastric mucosa during upper endoscopy is poor in routine practice (21). Another limitation is that in 7 cases, the Sydney protocol was not strictly applied, since only 4 biopsies were obtained (2 from the antrum and 2 from the corpus). However, in all other cases, the number of biopsies per endoscopy was higher than recommended (mean of 9 per endoscopy), and thus, we believe that gastric mucosa was assessed accurately providing reliable data on the natural evolution of GPL. Finally, because of the retrospective nature of this study, some parameters could not be taken into account, such as smoking habits or family history of GC. Besides, in some patients, assessment of *H. pylori* status was based on histology only, which could potentially lead to false-negative results. One other point is that ethnic factors may be involved in gastric carcinogenesis and that this “Low GC incidence area” population may be heterogeneous. However, French legislation do not allow to collect data on the ethnicity of the patients, and we were not able to provide information according to this criteria.

In conclusion, despite all these limitations, our study is the longest follow-up study of GPL in France, in a low-GC incidence European country, and it reliably shows that antrum-limited IM of incomplete type is associated with the highest risk of progression to GC. It also shows that IM of low OLGIM stage may regress after *H. pylori* eradication, after sufficiently long time. However, most GPL remain stable over the time, and the precise characterization of IM (type, OLGIM stage) should help to better identify the patients the most susceptible to benefit from surveillance.

To the best of our knowledge, this is the first study focusing of the evolution of the GPL in France. The major strengths of this study are the long follow-up period, the access to the individual data of patients treated in the same center, and the application of strict criteria dedicated to the investigation of GPL evolution, allowing for a precise analysis at a patient level.

Patients with antrum-limited IM are at risk to develop GC, especially in case of incomplete IM, indicating that these patients should be offered an appropriate surveillance. We also showed that the regression of IM is possible but after a sufficiently long time. Further studies, using targeted biopsies following the progress in magnifying endoscopy, and assessing the whole gastric mucosa status with non invasive markers, avoiding the risk of sampling error, will be useful for the surveillance of patients with GPL in the future.

## CONFLICTS OF INTEREST

**Guarantor of the article:** Tamara Matysiak-Budnik, MD, PhD.

**Specific author contributions:** Jean François Mosnier, MD, PhD, and Tamara Matysiak-Budnik, MD, PhD, share senior authorship. N.C., M.P., and T.M.-B.: contributed to the conception and design of the study, analysis and interpretation of data, drafted the manuscript, and revised it critically. J.F.M.: performed histopathological analysis of all biopsy specimens, contributed to analysis of data, and revised critically the manuscript. M.L.: performed statistical analyses. L.Q., Y.T., A.B., E.C., and E.C.: revised critically the manuscript. All the authors approved the final version of the manuscript.

**Financial support:** None to report.

**Potential competing interests:** None to report.

**Study Highlights**

**WHAT IS KNOWN**

✓ Patients with gastric precancerous lesions (atrophic gastritis and intestinal metaplasia) have an increased risk of gastric cancer.
✓ Data on the risk factors and time for progression of these lesions are scanty.

**WHAT IS NEW HERE**

✓ Patients with antrum-limited, incomplete-type intestinal metaplasia are at risk of progression to gastric cancer and should be offered surveillance.
✓ Progression of intestinal metaplasia is observed after a relatively long period (mean delay of 62 months in this study).
✓ Intestinal metaplasia does not appear always as a “point of no return” since it may regress in some cases after sufficiently long period (mean delay of 90 months in this study).

## Supplementary Material

SUPPLEMENTARY MATERIAL
