# Supplementary material for: Long-Term Follow-up of Gastric Precancerous Lesions in a Low GC Incidence Area
Source: Clin Transl Gastroenterol. 2020 Dec 3;11(12):e00237. doi: 10.14309/ctg.0000000000000237 (PMC7714054; doi:10.14309/ctg.0000000000000237)
Supplement: SUPPLEMENTARY MATERIAL [file ct9-11-e00237-s001.docx]

| **Initial GPL** | **Final GPL** | | | | | | | |
| --- | --- | --- | --- | --- | --- | --- | --- | --- |
|  | Normal | SG | AG | IM | LGD | HGD | Cancer |  |
| AG n=9 | 1 | 3 | 1 | 4 | 0 | 0 | 0 |  |
| IM n=195 | 14 | 24 | 4 | 140 | 8 | 1 | 2 |  |
| LGD n= 44 | 2 | 2 | 2 | 18 | 19 | 0 | 1 |  |
| HGD n=6 | 0 | 0 | 1 | 3 | 0 | 1 | 1 |  |

**Supplementary table 1.** Evolution of histologic findings between the initial and the last follow-up endoscopy. AG: Atrophic gastritis, HGD: High grade dysplasia, IM: Intestinal metaplasia, LGD: Low grade dysplasia, NAG: Non-atrophic gastritis

Most of the patients (161/254, 63.4%) presented the same GPL at first and last endoscopy, whereas in 74 patients (30%) the final lesion was less severe. Among them, 24 patients had LGD initially and in 18 cases, a regression was observed without any endoscopic or surgical intervention. Conversely, the 4 patients with initial HGD had an endoscopic resection of a visible lesion. Seven patients (2.8%) developed a GC. Among the 14 patients with IM located in the antrum only, two developed GC (OLGIM I and IV), two progressed to HGD and 10 to LGD. All six patients who had extensive (antrum and corpus) IM at initial diagnosis, had LGD at final endoscopy.

At initial endoscopy, *H. pylori* infection was found in 43% of patients overall (103 out of 240, missing data n=14 patients), and in 41.1%, 44.7% and 33.3% of patients with less severe, stable and more severe lesion at final endoscopy, respectively. Although all the patients have had eradication treatment prescribed according to the standard management in our center, persistent infection was found by histology in 17.6% of patients overall (42 out of 239, missing data =15) and in 10.1%, 22.1% and 6.3% of patients with less severe, stable and more severe lesion at final endoscopy, respectively.
